# A micromechanical study on the effects of precipitation on the mechanical properties of CoCrFeMnNi high entropy alloys with various annealing temperatures

**DOI:** 10.1038/s41598-023-30508-z

**Published:** 2023-02-28

**Authors:** Chang-Wei Huang, Pei-Ying Su, Chi-Hua Yu, Chia-Ling Wang, Yu-Chieh Lo, Jason Shian-Ching Jang, Hsuan-Teh Hu

**Affiliations:** 1 Department of Civil Engineering, Chung Yuan Christian University, No. 200, Zhongbei Rd, Taoyuan City, 32023 Taiwan; 2 Department of Civil Engineering, National Cheng Kung University, No.1, University Road, Tainan City 701, Taiwan; 3 Department of Engineering Science, National Cheng Kung University, No. 1, University Road, Tainan City, 701 Taiwan; 4 Department of Materials Science and Engineering, National Yang Ming Chiao Tung University, No. 1001, University Road, Hsinchu, 300 Taiwan; 5 Department of Mechanical Engineering, National Central University, No. 300, Zhongda Rd., Taoyuan, 320317 Taiwan; 6 Institute of Material Science and Engineering, National Central University, No. 300, Zhongda Rd., Taoyuan, 320317 Taiwan; 7 Department of Civil and Disaster Prevention Engineering, National United University, NO. 2, Lien Da, Miao-Li, 36063 Taiwan

**Keywords:** Metals and alloys, Computational methods

## Abstract

The CoCrFeMnNi high entropy alloys remain an active field over a decade owing to its excellent mechanical properties. However, the application of CoCrFeMnNi is limited because of the relatively low tensile strength. Here we proposed a micromechanical model which adopted from the theory of dislocation density to investigate the strengthening mechanisms of precipitation of chromium-rich non-equiatomic CoCrFeMnNi alloy. The microstructures of CoCrFeMnNi were obtained directly from SEM-BSE images with different annealing temperatures. The proposed framework is validated by comparing simulations with experiments of uniaxial tensile tests on the CoCrFeMnNi alloys under different annealing temperatures. The stress–strain curves indicate that the precipitate has greater influence on post-yield hardening than the initial yielding strength. In addition, we identified that the particle distribution, controlled by the average size of the particle and the volume fraction of precipitation, can significantly enhance the strengthening effect. The numerical results indicate that HEAs with a precipitate distribution closer to a normal distribution and with smaller average size will tend to have higher strength and ductility.

## Introduction

High entropy alloys (HEAs) have attracted a great deal of attention in recent years due to their excellent mechanical properties, such as good ductility, superior fatigue properties, and high strength, thermal stability, and wear resistance^1–6^. The name refers to the new design philosophy of alloying by introducing more metal elements. The most well-known HEA is the equimolar ratio CoCrFeNiMn alloy, also known as the Cantor alloy^7,8^. Although it has a complicated chemical composition, it forms a simple solid solution phase that gives it extraordinary ductility and fracture toughness at elevated temperatures (approximately 900 °C). The yield strength and tensile strength of CoCrFeNiMn, however, is relatively low, and this imbalanced mechanical performance limits its potential applications^8–10^. Therefore, it is crucial to improve its strength without introducing additional brittleness.

The strength of CoCrFeMnNi high entropy alloy is affected by the phase of its microstructures. Several relevant factors influence its yield strength and post-yield behavior, such as the components of the HEA, lattice distortion, the grain distribution, and the presence of defects (*e.g.,* doping, precipitation, and dislocation). These mechanisms can be expressed as the intrinsic strength, solid solution strengthening, grain boundary strengthening, and precipitation strengthening of an HEA.

Of these strengthening mechanisms, precipitation is relatively difficult to study because the characteristic length of precipitation is smaller than the observed microstructure. Precipitation has been found in Cantor alloys after annealing at temperatures below 800 °C^11,12^. Multicomponent solid solutions that have stabilized from high configurational entropy may decompose between 600 and 800 °C due to dislocations and local chemical perturbations at their grain boundaries. This discovery overturned the previous conceptualization of high-entropy alloy CoCrFeMnNi as a single-phase HEA.

Second phase precipitation affects the grain growth process in the FCC matrix. Recent studies have shown that in CoCrFeNiMn alloys, second-phase precipitates can effectively hinder grain growth and form fine-grained structures^11,13,14^. The precipitation strengthening effect produced by the σ-phase can effectively improve the yield strength, tensile strength, and strain hardening rate^15,16^. Therefore, studies on the precipitation of the second phase have been conducted with the aim of enhancing this effect, for example, by adding different elements such as V, Ti, Al, and C^17–19^. The phase stability and kinetics of precipitates under different heat treatment processes have also been studied^20,21^, along with the effect of precipitates on the yield strength, ductility, and grain growth of CoCrFeMnNi alloys^15^.

Despite various experimental studies that have suggested that precipitation plays a significant role in yield strength and post-yield hardening, there still limited theoretical study has quantitively investigated the relationship^22^. Therefore, a mesoscale model is urgently needed to elucidate the strengthening mechanisms of CoCrFeMnNi to improve its yield strength and post-yield behavior. Crystal plasticity finite element method (CPFEM) has been applied to study the plastic anisotropy and work hardening of aluminum alloys^23^. CPEFM integrates the crystal plasticity theory with the finite element method and considers the interaction between the internal grains and the complex boundary conditions. This method enables the study of grain size effects and deformation behavior at complex stress fields and high strain rates. To further extend the ability of CPFEM to deal with precipitation, the hardening model used in this study utilized two dislocation densities: geometrically necessary dislocation density *ρ*_*g*_ and statistically stored dislocation density *ρ*_*s*_. Dislocation density-based CPFEM enables us to describe deformation on the microscale, expressing the effects of dislocation growth, reduction relationships, and precipitation.

The purpose of this study is to conduct a comprehensive investigation of the mechanical properties of CoCrFeMnNi high-entropy alloys at different annealing temperatures. The alloys used in the study were tested for tensile strength at room temperature and the deformation mechanism of the alloy was simulated with controlled slip differentials. The simulation was based on the results of the tensile test after annealing for one hour at three different annealing temperatures: 1073 K, 1173 K, and 1273 K. The simulation stress–strain curve is verified using the results of the tensile test of the chromium-rich non-equiatomic CoCrFeMnNi alloy studied by Cho et al.^24^, which matches the mechanical properties of the actual material.

Special attention is paid to dislocation, the precipitation strengthening effect, and the effect of precipitate particle size and distribution. The influence of precipitation strengthening on post-yielding behavior and the relationship between dislocation density and the stress–strain curves are also discussed in great detail. The size and distribution of the precipitates are considered for the parameter calculation. Two types of particle size distributions are simulated: normal distribution and real distribution, which were obtained from an image analysis conducted using ImageJ. The simulation results are compared and the effect of different particle distributions on the hardening behavior of the alloy are discussed.

## Materials and methods

**Figure 1:**
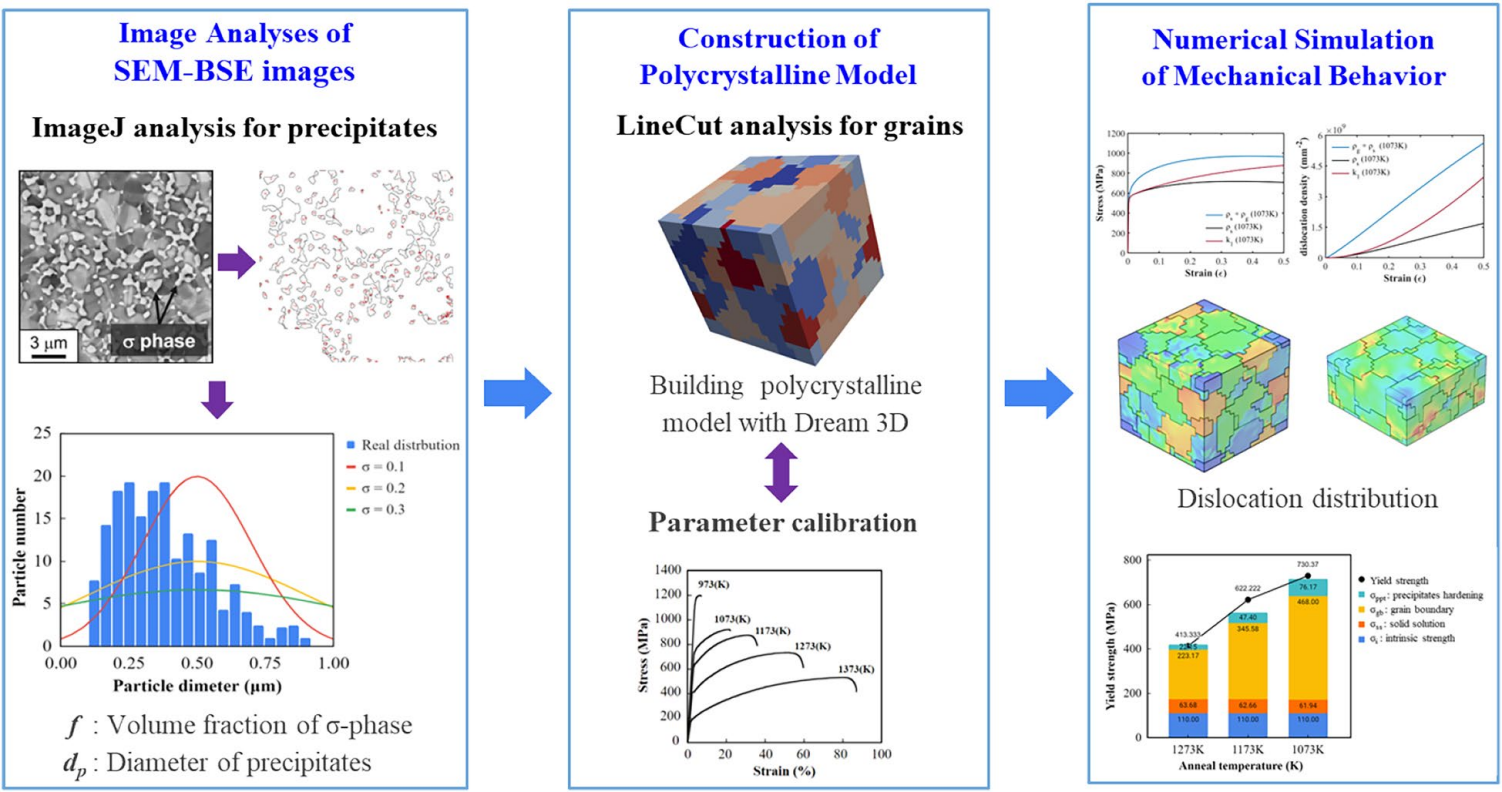
Analysis procedure for the study of the strengthening mechanisms of the Cr-rich CoCrFeMnNi alloy using dislocation density-based CPFEM.

The details of the numerical framework will be introduced in the following subsections. Analysis procedure of the proposed computational framework is depicted in Fig. 1. First, in order to obtain microstructure, we exploited an open sourced software, ImageJ^25^, to process SEM-BSE images of the CoCrFeMnNi specimens for the tensile tests^24^. We then constructed polycrystalline models using Dream.3D according to the microstructures. The mechanical behavior of the CoCrFeMnNi specimens was analyzed based on the dislocation density-based crystal plasticity finite element method (CPFEM) using Abaqus user materials (UMAT). Numerical simulations of the tensile tests were performed to calibrate all the necessary parameters. Finally, we used the calibrated model to predict the mechanical behavior of the CoCrFeMnNi specimens with different annealing temperatures.

### Material and mechanical properties

**Figure 2:**
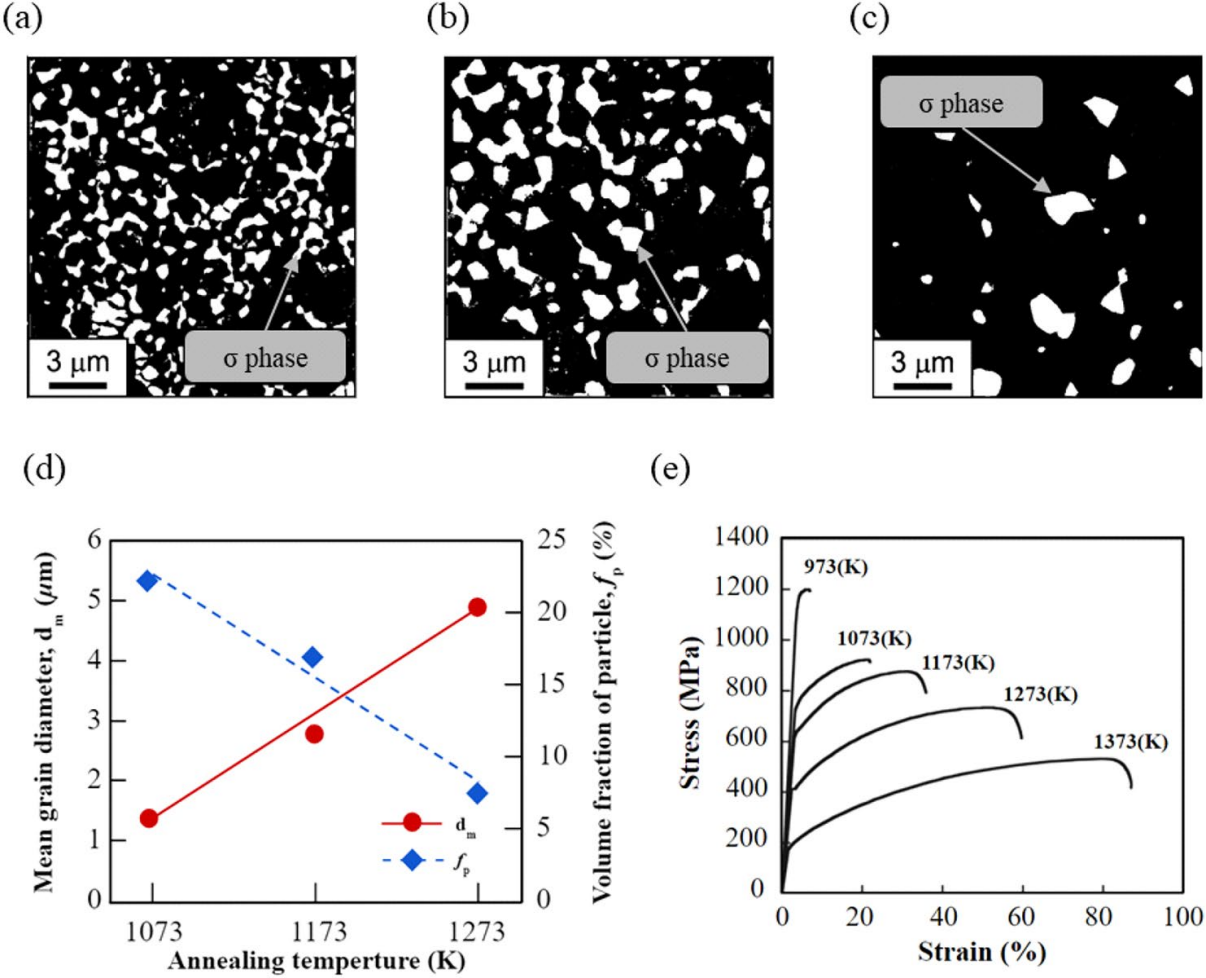
SEM-BSE images of the Co_20_Cr_25_Fe_20_Mn_20_Ni_15_ alloy annealed at (**a**) 1073 K, (**b**) 1173 K, and (**c**) 1273 K for one hour after 90% cold rolling. (**d**) Variation in the mean grain diameter and the volume fraction of the 25Cr as a function of annealing temperature. (**e**) The stress strain curves of the 25Cr annealed at different temperatures for one hour. (The raw data of the SEM-backscattered electron (BSE) images and the stress–strain curves for the CoCrFeMnNi alloy annealed at different temperatures were obtained from Cho et al.^24^ The data was extracted and reproduced in the present study).

In this study, the chromium-rich high entropy alloy Co_20_Cr_25_Fe_20_Mn_20_Ni_15_ which was produced from high purity Co, Cr, Fe, Mn and Ni by plasma arc melting in an Ar atmosphere is investigated. The master ingots were then cold rolled at room temperature to a thickness reduction of 90% and then recrystallised by annealing at 1073–1373 K for one hour in an Ar atmosphere followed by water quenching^24^. The SEM-BSE images show a fully recrystallized coarse-grained microstructure without the σ-phase after annealing at a high temperature of 1373 K; however, after annealing at the lower temperature of 973 K, the σ-phase was observed to precipitate, while the recrystallization of the face-centered crystal (FCC) structure matrix was not complete. The fully recrystallized fine FCC matrix and the fine σ-phase precipitates could be observed in the alloy after annealing at 1073–1273 K for one hour, as shown in Fig. 2a–c. Figure 2d shows that the grain size decreases, and the volume fraction of σ-phase particles increases with decreasing annealing temperature. These results imply that the precipitation of the σ-phase plays an important role in the grain refinement of the alloys.

The mechanical properties of the CoCrFeMnNi alloys were evaluated by tensile tests at room temperature with a strain rate of 1.7 × 10^–4^ s^–1^ ^24^. The tensile test specimens with gauge dimensions of 5.0 × 1.5 × 1.0 mm were cut from the annealed plates of the alloys by an electro-discharge machining^24^. The stress–strain curves obtained from the tensile test of the CoCrFeMnNi samples with different annealing temperatures are shown in Fig. 2e^24^. The experimental results show that the strength of the thermally treated alloys increases with the decrease in the annealing temperature. The strength of the alloys with precipitates was also significantly higher than that of alloys without precipitates.

### Yield strength

An important factor in the crystal plasticity finite element method is the yield strength. The initial yield strength of an alloy at room temperature, $\sigma_{0}$, is determined by considering the following mechanisms: intrinsic strength, solid solution strengthening, grain boundary strengthening, and precipitation strengthening^26^. Therefore, the initial yield strength of an alloy can be expressed as:

1 $$\sigma_{{0}} = \sigma_{is} + \sigma_{ss} + \sigma_{gb} + \sigma_{ps}$$

where $\sigma_{is}$ , $\sigma_{gb}$, $\sigma_{ss}$, and $\sigma_{ps}$ represent the yield strength contribution from the intrinsic strength, grain boundary, solid solution, and precipitation, respectively.

According to Vegard's law^27^, the intrinsic strength, which is also known as the lattice friction strength, can be obtained from:

2 $$\sigma_{is} = \sum\limits_{j} {X_{j} \sigma_{0j} }$$

where *X*_*j*_ and $\sigma_{0j}$ are the atomic fraction and friction stress of the dislocation motion for the *j*-th element. In addition, the solid solution strengthening of an HEA resulting from the interaction of dislocations with solute atoms can be calculated by^28,29^:

3 $$\sigma_{ss} = AG\left( {\sum\limits_{j} {\varepsilon_{j}^{2} X_{j} } } \right)^{2/3}$$

where *A* is a dimensionless parameter related to the material, *G* is the shear modulus of the HEA material, and $\varepsilon_{j}$ represents the individual contribution to the overall yield strength of the *j*-th component element in the HEA, which can be estimated by:

4 $$\varepsilon_{j} = (\eta_{j}^{2} + a_{p}^{2} \delta_{j}^{2} )^{1/2}$$

where $a_{p}$ is the plastic deformation constant determined by the type of dislocation, and $\eta_{j}$ and $\delta_{j}$ are the elastic modulus misfit and atomic size misfit between the *j*-th atom and other atoms, respectively. These two misfit parameters are calculated as:

5a $$\eta_{j} = \frac{13}{{12}}\sum\limits_{i} {X_{i} \cdot \frac{{2 \cdot (G_{j}^{(0)} - G_{i}^{(0)} )}}{{(G_{j}^{(0)} + G_{i}^{(0)} )}}}$$

5b $$\delta_{j} = \frac{13}{{12}}\sum\limits_{i} {X_{i} \cdot \frac{{2 \cdot (R_{j}^{(0)} - R_{i}^{(0)} )}}{{(R_{j}^{(0)} + R_{i}^{(0)} )}}}$$

where *G*^(0)^ denotes the shear modulus of the pure component and *R*^(0)^ denotes half of the average distance between the nearest atoms of the pure component in the lattice of the multicomponent alloy.

Furthermore, the contribution of grain boundary strengthening to the yield strength for crystalline materials can be obtained from the Hall–Petch equation:

6 $$\sigma_{ss} = k \cdot d_{g}^{ - 1/2}$$

where *k* and *d*_*g*_ represent the Hall–Petch slope and the mean grain size of the considered alloy, respectively. Finally, the contribution of precipitation strengthening to the yield strength can be expressed as^30^:

7 $$\sigma_{ps} = \frac{M}{{b^{2} \sqrt G }}\sqrt {\frac{{r_{m} \sum {N_{j} } }}{0.5}} (F_{m} )^{3/2}$$

where *M* is the Taylor factor, *b* is the Burgers vector, and *r*_*m*_ and *N*_*j*_ represent the mean particle size and number of *j*-th particles per unit volume, respectively. *F*_*m*_ is the mean obstacle strength and can be obtained by^31^:

8a $$F_{m} = \frac{{\sum {N_{j} F_{j} } }}{{\sum {N_{j} } }}$$

8b $$F_{j} = \left\{ \begin{gathered} 2\beta Gb^{2} (\frac{{r_{j} }}{{r_{c} }}){\text{ for }}r_{j} < r_{c} \hfill \\ 2\beta Gb^{2} {\text{ for }}r_{j} \ge r_{c} \hfill \\ \end{gathered} \right.$$

where β is a constant close to 0.5 and *F*_*j*_ is the obstacle strength, which is determined by the radius for the *j*-th precipitate. If the precipitate radius, *r*_*j*_, is less than the critical radius for shear *r*_*c*_, it is a shearable precipitate and the strength contribution will be reduced with the size of the critical radius *r*_*c*_.

### Crystal plasticity finite element method

The micromechanics of alloys can be described by the theory of crystal plasticity^32,33^. In the crystal plasticity model, the deformation of the crystalline crystal is described by the deformation gradient and the velocity gradient. The deformation of the crystal is divided into an elastic part and a plastic part. The former obeys Hooke's law, and the latter is dominated by the slip system in the crystal.

Considering the deformation gradient from the initial position **X** to the current position **x**, the deformation gradient **F** can be defined as:

9 $${\mathbf{F}} = \frac{{\partial {\mathbf{x}}}}{{\partial {\mathbf{X}}}} = \frac{{\partial {\mathbf{u}}}}{{\partial {\mathbf{X}}}} + {\mathbf{I}}$$

where **u** is the displacement vector and **I** is the second-order identity matrix. The deformation gradient can be further divided into the elastic part **F**^*e*^ and the plastic part **F**^*p*^ by:

10 $${\mathbf{F}} = {\mathbf{F}}^{e} {\mathbf{F}}^{p}$$

Additionally, the corresponding velocity gradient is defined as:

11 $${\mathbf{L}} = \frac{{\partial {\mathbf{v}}}}{{\partial {\mathbf{X}}}} = {\dot{\mathbf{F}}\mathbf{F}}^{ - 1}$$

where a dot is the partial derivative with respect to time. The velocity gradient can also be rewritten in terms of **F**^*e*^ and **F**^*p*^ as:

12 $${\mathbf{L}} = {\dot{\mathbf{F}}\mathbf{F}}^{ - 1} = {\dot{\mathbf{F}}}^{e} ({\mathbf{F}}^{e} )^{ - 1} + {\mathbf{F}}^{e} {\dot{\mathbf{F}}}^{e} ({\mathbf{F}}^{p} )^{ - 1} ({\mathbf{F}}^{e} )^{ - 1}$$

The plastic deformation of the crystal is primarily the result of the sliding of the slip systems caused by the resolved shear stress. Therefore, the plastic velocity gradient of the crystal deformation can be expressed as the sum of the actions of each group of sliding systems in the crystal:

13 $${\mathbf{L}}^{p} = {\dot{\mathbf{F}}}^{p} ({\mathbf{F}}^{p} )^{ - 1} = \sum\limits_{\alpha = 1}^{n} {\dot{\gamma }^{\alpha } {\mathbf{s}}^{\alpha } \otimes {\mathbf{m}}^{\alpha } }$$

where $\dot{\gamma }^{\alpha }$ is the shear strain rate of the α-th slip system, and **s**^α^ and **m**^α^ represent the α-th unit slip direction vector and the α-th unit slip plane normal vector, respectively.

The constitutive law model of the crystal plastic finite element method can be expressed as^34,35^:

14 $${\mathbf{T}}^{e} { = }{\mathbf{CE}}^{e}$$

where **C** is the fourth-order elasticity tensor and **T**^*e*^ and **E**^*e*^ are the conjugate pair of stress and strain tensors, respectively, which are defined by:

15 $${\mathbf{T}}^{e} { = (}{\mathbf{F}}^{e} )^{ - 1} [det({\mathbf{F}}^{e} ){\mathbf{T}}]({\mathbf{F}}^{e} )^{ - T}$$

16 $${\mathbf{E}}^{e} { = }\frac{1}{2}[({\mathbf{F}}^{e} )^{T} {\mathbf{F}}^{e} - {\mathbf{I}}]$$

where **T** is the Cauchy stress of the grain. The resolved shear stress $\tau^{\alpha }$ on the α-th slip surface can be approximated by:

17 $$\tau^{\alpha } \approx {\mathbf{T}}^{e} \cdot {\mathbf{s}}_{0}^{\alpha } \otimes {\mathbf{m}}_{0}^{\alpha }$$

To describe the plastic behavior of a material, it is necessary to define the flow rule and the hardening rule. The flow rule is used to describe the shear deformation rate caused by the dislocation slip in a specific slip system. According to the Peirce-Asaro-Needleman model^36^, the shear deformation rate of a slip system can be defined by the flow rule:

18 $$\dot{\gamma }^{\alpha } = \dot{\gamma }_{0} {(}\left| {\frac{{\tau^{\alpha } }}{{\tau_{c}^{\alpha } }}} \right|{)}^{N} {\text{sign}}(\frac{{\tau^{\alpha } }}{{\tau_{c}^{\alpha } }})$$

where $\dot{\gamma }_{0}$ is the initial shear strain rate, $\tau_{c}^{\alpha }$ is the current critical resolved shear stress (resolved shear strength) of the α-th slip system, *N* is the strain rate sensitivity coefficient, and the sign function indicates the direction of the slip. It should be noted that the movement of the slip in the α-th slip plane is activated when $\tau_{ }^{\alpha }$ is larger than $\tau_{c}^{\alpha }$.

Based on the dislocation hardening models^37–39^, the master hardening curve for the critically resolved shear stress can be given by:

19 $$\tau_{c} = \tau_{0} + aGb\sqrt {\rho_{s} + \rho_{g} }$$

where $\tau_{0}$ is the initial yield shear strength. In this study, $\tau_{0}$ is equal to $\sigma_{y}$/*M* with the Taylor factor *M* = 3.06. In addition, *ρ*_*s*_ and *ρ*_*g*_ represent the statistically stored and the geometrically necessary dislocation densities, respectively, *a* is a constant and is set to be 0.3 in this study, and *G* and *b* are the shear modulus at room temperature and the Burgers vector of the CoCrFeMnNi alloy, respectively.

The statistically stored dislocation density represents the growth and recovery of dislocations. The evolution of the statistically stored dislocation density can be described as follows based on the Kocks-Mecking model^40,41^:

20 $$\frac{{d\rho_{s} }}{{d\varepsilon^{p} }} = (k_{1} \rho_{s}^{1/2} - k_{2} \rho_{s} )$$

where *k*_1_ describes the accumulation of the statistically stored dislocation during deformation and is a constant for the material under consideration. It can be obtained by:

21 $$k_{1} = (\frac{G}{{G_{0} }})^{2} \cdot \frac{1}{100\alpha }$$

where *G*_0_ is the shear modulus of CoCrFeMnNi at 0 K and *α* is the thermal activation constant of the Taylor relation. Furthermore, *k*_2_ is the dynamic recovery term of the statistically stored dislocation and can be obtained by fitting the experimental results.

On the other hand, the evolution of the geometrically necessary dislocation density can be described as^39^:

22 $$\frac{{d\rho_{g} }}{{d\varepsilon^{p} }} = \frac{1}{bL}[1 - (\frac{{\rho_{g} }}{{\rho_{g}^{sat} }})^{m} ]$$

where *L* is the average slip distance of Orowan precipitates and $\rho_{g}^{sat}$ is the density of geometrically necessary dislocations at saturation. The value of parameter *m* needs to be set sufficiently high and we use *m* = 10 in this study^39^. The average slip distance of Orowan precipitates, *L*, can be calculated by^39^:

23 $$L = k_{4} \lambda_{g,0}$$

where *k*_4_ and $\lambda_{g,0}$ represent a constant and the characteristic geometric slip distance associated with the Orowan particles, respectively. In this study, the average slip distance of the Orowan precipitates, *L*, is assumed to equal $\lambda_{g,0}$ for simplicity. In addition, $\lambda_{g,0}$ can be estimated by the particle size distribution as^39^:

24 $$\lambda_{g,0} = \frac{1}{{8\sum\limits_{i} {r_{i}^{2} N_{i} } }},{\text{ for }}r_{i} > r_{c}$$

where *r*_*i*_ and *r*_*c*_ represent the *i*-th radius of the precipitates and the critical radius of the precipitates, respectively. According to Eq. (24), only when a precipitate radius is larger than the critical radius do non-shearable precipitates have a strengthening effect on the alloy. *N*_*i*_ is the number of precipitates with a specific radius *r*_*i*_ per unit volume.

It should be noted that the geometrically necessary dislocation density does not increase indefinitely^39^ and $\rho_{g}^{sat}$ is its upper limit. It can be expressed as a relationship with the average slip distance *L* and the volume fraction *f*_*p*_ by^39^:

25 $$\rho_{g}^{sat} = \frac{{k_{5} }}{{f_{p} bL}},{\text{ for }}r_{i} > r_{c}$$

where *k*_5_ is constant approximately equal to 1.0 and *f*_*p*_ is the volume fraction of the precipitates.

### Analysis preprocessing

SEM-BSE images of CoCrFeMnNi alloys with three different annealing temperatures (from 1073 to 1273 K) were analyzed using the ImageJ software to obtain the precipitate data. First, a threshold analysis was conducted to identify the precipitates in the images according to a prespecified volume fraction *f*_*p*_. Then particle analysis was conducted to calculate the area and number of precipitates in the selected area. Based on the spherical precipitate assumption, the obtained data were converted to the radius of the precipitates *r*_*i*_*,* the number of precipitates with a specific radius *r*_*i*_ per unit volume *N*_*i*_, and the mean radius *r*_*m*_ of the precipitates, as shown in Table 1.

**Table 1:** Post-processing of experimental data (ImageJ analysis).

	1273 K	1173 K	1073 K
SEM-BSE binary image			
ImageJ			
Particle distribution			
*f*_*p*_ (%)	7.6%	16.8%	22%
*r*_*m*_ (*μ*m)	0.4776	0.3510	0.2499

It is worth noting that an SEM-BSE image can only provide the precipitate information for a certain cross-section of the specimen. As a result, precipitate information for the whole specimen may not be available.

Two methods were used to calculate the number of precipitates per unit volume, *N*_*i*_, with radius *r*_*i*_ for a pre-specified volume fraction *f*_*p*_. In the first method, we assumed that the precipitate size distribution satisfied a normal distribution and the mean radius of precipitates was obtained from image analyses. Then the number of particles *N*_*i*_ with radius* r*_*i*_ per unit volume could be obtained from:

26 $$N_{i} = \frac{{f_{p} \cdot \phi (r_{i} )}}{{\sum\limits_{i} {\frac{4\pi }{3}r_{i}^{3} \cdot \phi (r_{i} )} }}$$

where *ϕ*() is the probability density function for the normal distribution.

In the second method, we assumed that the precipitate information obtained from the SEM-BSE image analyses represented the real precipitate size distribution. Then the number of precipitates *N*_*i*_ with radius *r*_*i*_ per unit volume could be calculated by:

27 $$N_{i} = \frac{{f_{p} \cdot P_{i} }}{{\sum\limits_{i} {\frac{4\pi }{3}r_{i}^{3} \cdot P_{i} } }}$$

where *P*_*i*_ represents the number of precipitates with radius *r*_*i*_ in the SEM-BSE image.

The corresponding grain size distributions of the CoCrFeMnNi specimens with the three different annealing temperatures were obtained by analyzing the SEM-BSE images using the LineCut method^42^. Subsequently, polycrystalline models were constructed using the open-source Dream.3D software that were consistent with the real grain size and distribution of the CoCrFeMnNi specimens at the different annealing temperatures^43^. The grain orientations of these representative volume element (RVE) models were assigned to random Euler angles through a Python script to conform to the randomness of the experiments. In addition, periodic boundary conditions (PBCs) were applied to these RVE models to reduce the boundary effect in our simulations. The number of solid elements in each RVE model is given by 20^3^ (8000). Depending on the number of grains in each model, the element resolutions of the RVE models were set to 0.2, 0.4, and 0.7 for each side length for the CoCrFeMnNi specimens at the three respective annealing temperatures. Detailed settings of these RVE models are shown in Table 2.

**Table 2:** Polycrystalline models (Dream 3D analysis).

	1273 K	1173 K	1073 K
Polycrystalline model			
Grain size (*μ*m)	4.9	2.8	1.41
Grain number	41	42	43
Model size (*μ*m^3^)	2744	512	64
Elements	20^3^	20^3^	20^3^

### Model parameters

The initial yield strength of the CoCrFeMnNi alloys could be estimated by Eq. (1). The values of the related material parameters of each compositional element are listed in Table 3. In addition, the Burgers vector, *b*, and the critical radius for shear, *r*_*c*_, are given as 0.25 nm^44^ and 1.7 nm^45^ in the literature, respectively. Given the sizes of the precipitates and grains of the CoCrFeMnNi specimens with different annealing temperatures, we can calculate the theoretical yield strength, $\sigma_{y}$, for the CoCrFeMnNi specimens as 420.72 MPa, 559.52 MPa, and 714.07 MPa for annealing temperatures 1273 K, 1173 K, and 1073 K, respectively.

**Table 3:** Material parameters of each compositional element.

Parameter	Fe	Co	Ni	Cr	Mn
*X*_*j*_ (%)	20.5	20.5	15	24	20
*σ*_*0j*_ (MPa)	166	10	9.6	217	110
*G*_*j*_^(0)^ (GPa)	82	75	76	115	81
*R*_*j*_^(0)^ (nm)	0.156	0.152	0.149	0.166	0.161
$\eta_{j}$	− 0.0523	− 0.1481	− 0.1339	0.3094	0.0247
$\delta_{j}$	− 0.0098	− 0.0379	− 0.0595	0.0575	0.0244
$\varepsilon_{j}$	3.441	19.244	26.451	40.752	9.466

The face-centered cubic (FCC)-structured crystals have three independent elastic constants, which can be obtained from Young’s modulus *E* and Poisson’s ratio *v* as^46^:

28a $$C_{11} = \frac{E(1 - \nu )}{{(1 + \nu )(1 - 2\nu )}}$$

28b $$C_{12} = \frac{E\nu }{{(1 + \nu )(1 - 2\nu )}}$$

28c $$C_{44} = \frac{E}{2(1 + \nu )}$$

These three elastic constants were calculated using *E* = 202 GPa (from room temperature tensile tests) and *v* = 0.3 (from our assumption)^47,48^. In addition, the initial shear strain rate, $\dot{\gamma }_{0}$, is given by 1.0 × 10^–6^^49^.

**Figure 3:**
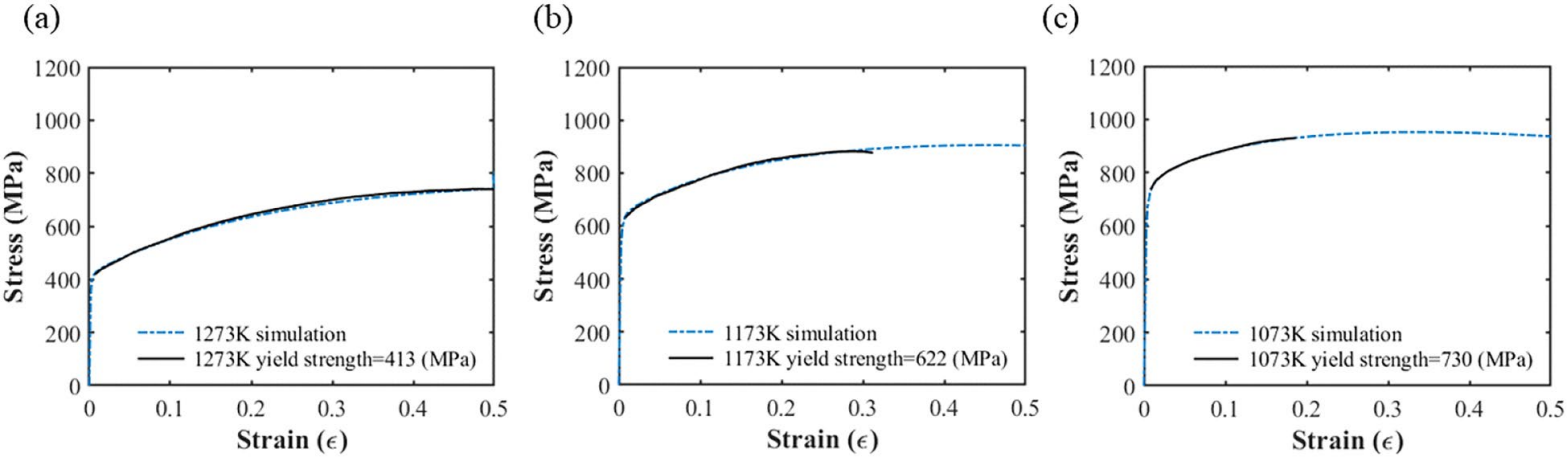
The stress strain curves for the CoCrFeMnNi specimens of different annealing temperatures obtained from numerical simulation: (**a**) 1273 K, (**b**) 1173 K, and (**c**) 1073 K.

Numerical simulations of the tensile tests were performed using the polycrystalline models and precipitate parameters to calibrate some of the model parameters. The strain rate was set at 10^–4^ s^-1^ in these tensile tests. The model parameters for the CoCrFeMnNi specimens of different annealing temperatures obtained by curve fitting are listed in Table 4. It is worth noting that the symbol *σ* represents the standard variation of the precipitate radius under the assumption of a normal distribution. The stress–strain curve obtained for the different samples is shown in Fig. 3, where the simulation and experimental results^24^ are represented by blue and black lines, respectively. From Fig. 3 it can be seen that the simulation results agree well with the experimental data, with *R*^2^ = 0.98.

**Table 4:** Model parameters obtained from curve fitting.

	1273 K	1173 K	1073 K
*N*	65	65	65
*k*_1_	1.13 × 10^5^	1.13 × 10^5^	1.13 × 10^5^
*k*_2_	0.65	1.2	1.6
*σ* (*μ*m)	0.148	0.133	0.200

## Results

### The strengthening mechanisms of initial yield strength

The contribution of each strengthening mechanism to the initial yield strength of the CoCrFeMnNi samples with different annealing temperatures is listed in Table 5. Since the concentrations of the compositional elements did not change, the intrinsic strength and the solid solution strength remained constant across annealing temperatures. Furthermore, the grain boundary strengthening mechanism dominated the initial yield strength for all annealing temperatures, since the mean grain size decreases from 4.9 to 1.41 μm as the annealing temperature decreases. As a result, the strengthening of the grain boundary increased according to the Hall–Petch effect. Furthermore, the contribution of precipitation strengthening increases with decreasing annealing temperature. For the CoCrFeMnNi specimens, the average radius *r*_*m*_ of the precipitates was larger than the critical radius *r*_*c*_. As a result, the mean obstacle strength, $\overline{F}$, remained constant across annealing temperatures but the precipitation strength varied due to variation in the number of particles per unit volume and the average radius of the particles.

**Table 5:** Contribution of each strengthening mechanism to the initial yield strength of the CoCrFeMnNi specimens with different annealing temperatures (unit: MPa).

	1273 K	1173 K	1073 K
*σ*_*is*_	110.14	108.03	107.61
*σ*_*ss*_	62.66	62.66	62.66
*σ*_*gb*_	223.17	345.58	468.00
*σ*_*ps*_	23.72	43.25	76.52
(*σ*_0_)_theory_	420.72	559.52	714.07
(*σ*_0_)_experiment_	413.33	622.22	730.37

### Dislocation densities

**Figure 4:**
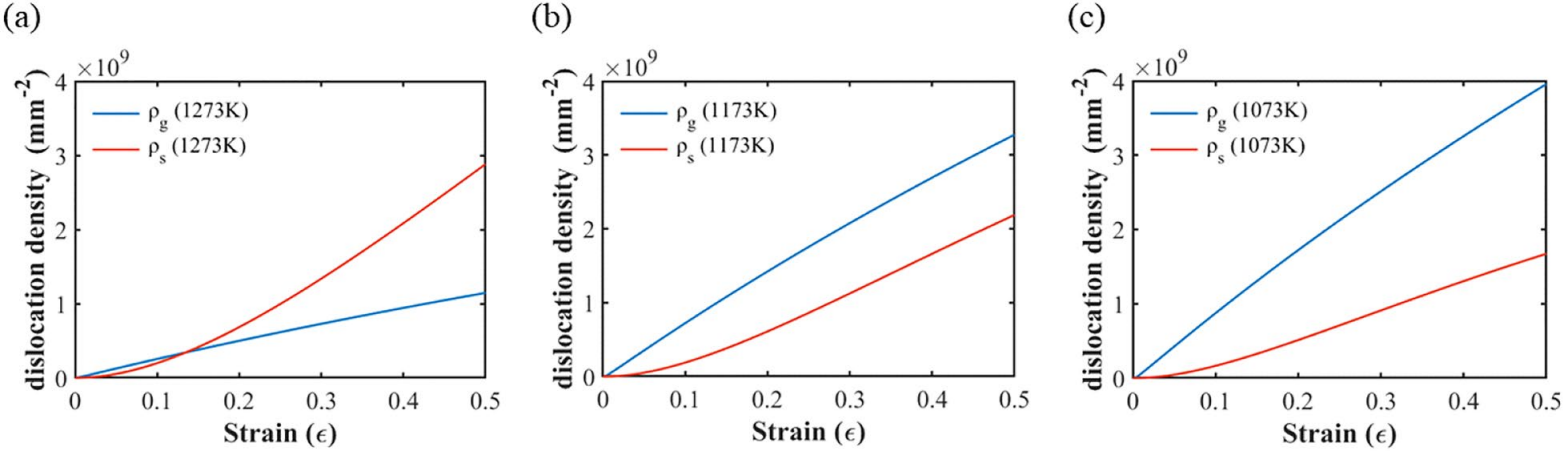
Evolution curves of the dislocation density for three annealing temperatures: (**a**) 1273 K, (**b**) 1173 K, and (**c**) 1073 K.

Figure 4a–c depicts the dislocation density for the three annealing temperatures from 1073 to 1273 K. The statistically stored dislocation density *ρ*_*s*_ and the geometrically necessary dislocation density *ρ*_*g*_ are represented by the red and blue lines, respectively. The geometrically necessary dislocation density *ρ*_*g*_ increases with decreasing annealing temperature. In contrast, the statistically stored dislocation density *ρ*_*s*_ increases with increasing annealing temperature. In the case of the 1273 K annealing temperature, *ρ*_*s*_ < *ρ*_*g*_ during the early deformation stage. With increasing deformation strain, *ρ*_*s*_ increased rapidly and became higher than *ρ*_*g*_. For lower annealing temperatures, *ρ*_*g*_ was higher than *ρ*_*s*_ during the tensile test.

Comparing the stress–strain curves with the dislocation density curves in Figs. 3 and 4, one can see that the strength of the CoCrFeMnNi specimens increases as the annealing temperature decreases. Meanwhile, *ρ*_*g*_ also increases but *ρ*_*s*_ decreases. The influence of the statistically stored dislocation density and the geometrically necessary dislocation density on the strengthening of the specimens with different annealing temperatures will be discussed in “The Influence of dislocation density on the stress–strain curve” section.

### Model parameters versus annealing temperature

**Figure 5:**
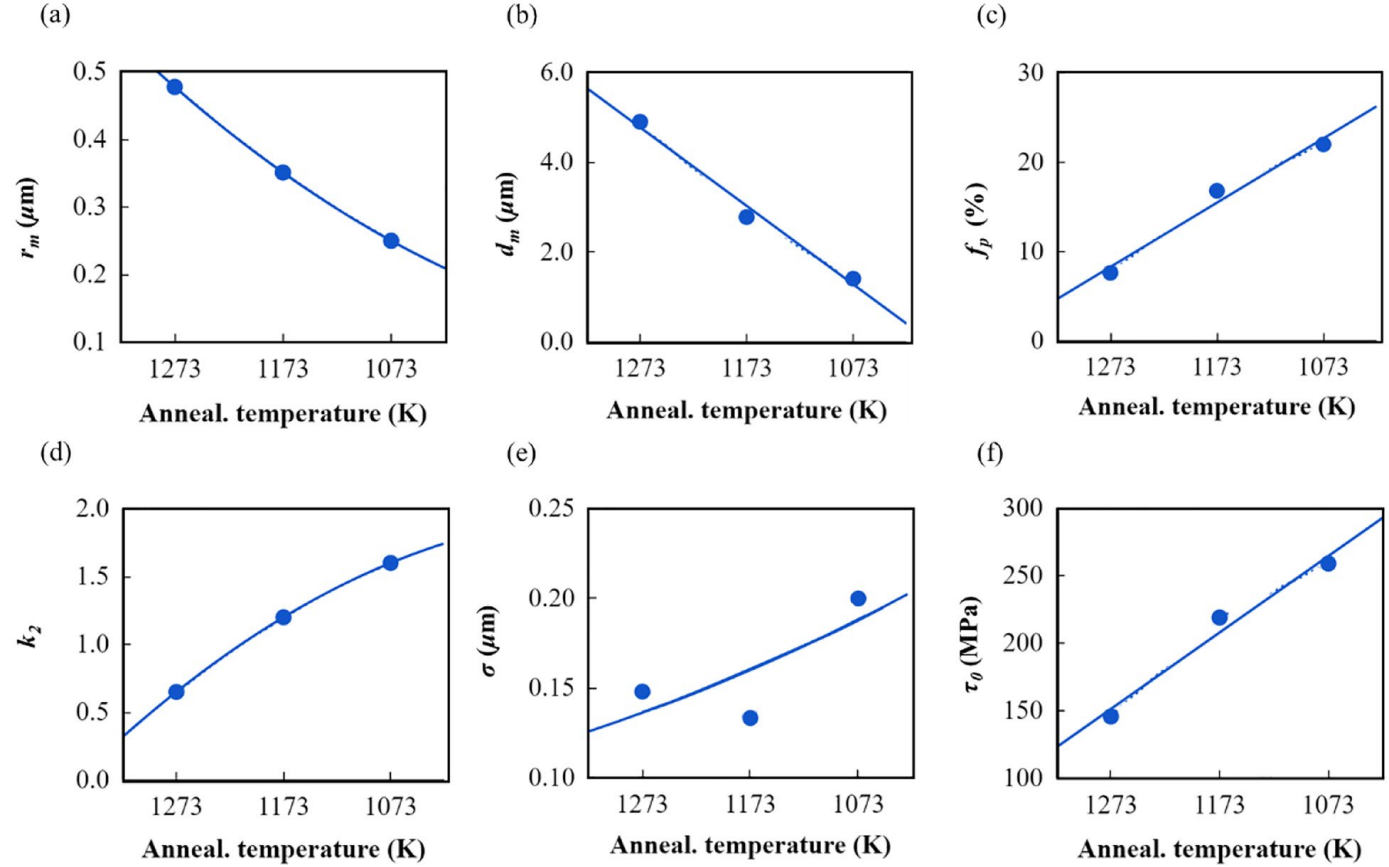
The relationships between the model parameters and annealing temperature: (**a**) mean radius of the precipitate; (**b**) mean size of the train; (**c**) volume fraction of the precipitates; (**d**) dynamic recovery parameter of dislocations stored statistically; (**e**) standard deviation of the precipitates with a normal distribution; and (**f**) initial shear strength of the yield.

Based on the parameter values listed in Tables 1 and 2 for the three annealing temperatures, we can construct the relationship between them, as shown in Fig. 5a–f. It can be found that both the precipitate particles and the grains become smaller as the annealing temperature decreases. The mean value of the precipitate radius, *r*_*m*_, and the mean value of the grain size, *d*_*g*_, can be described by (unit: *μ*m):

29a $$r_{m} (T) = 0.78 - 1.872 \times 10^{ - 3} \times T + 1.28 \times 10^{ - 6} \times T^{2}$$

29b $$d_{g} (T) = - 17.3138 + 0.01745 \times T$$

where *T* is the annealing temperature in terms of the absolute temperature.

In addition, the volume fraction of the precipitates (%), *f*_*p*_, and the model parameter, *k*_2_, which controlled the dynamic recovery behavior of the statistically stored dislocations, can be expressed as:

30a $$f_{p} (T) = 88.3 - 0.0624 \times T$$

30b $$k_{2} (T) = - 3.55 + 1.284 \times 10^{ - 2} \times T - 7.493 \times 10^{ - 6} \times T^{2}$$

The standard deviation *σ* (unit: *μ*m) of the precipitates with a normal distribution and the initial yield shear strength can be calculated by:

31a $$\sigma (T) = 181 \times Exp( - 0.0051 \cdot T) \times r_{m}$$

31b $$\tau_{0} (T) = 599.5 - 0.376 \times T.$$

## Discussion

### The Influence of dislocation density on the stress–strain curve

In order to investigate the influence of dislocation density on the stress–strain curve, different dislocation-based hardening rules were tested for the CoCrFeMnNi specimens. The three hardening rules were: (1) based on statistical stored dislocations and geometrically necessary dislocations, (2) based on statistical storage dislocations only (including accumulative and dynamic recovery), and (3) based on statistical storage dislocations only but with the accumulation term (*k*_1_) and without the dynamic recovery term (*k*_2_). It is worth noting that the hardening rule (2) can be interpreted as a special case of rule (1), which implies an alloy without precipitation.

**Figure 6:**
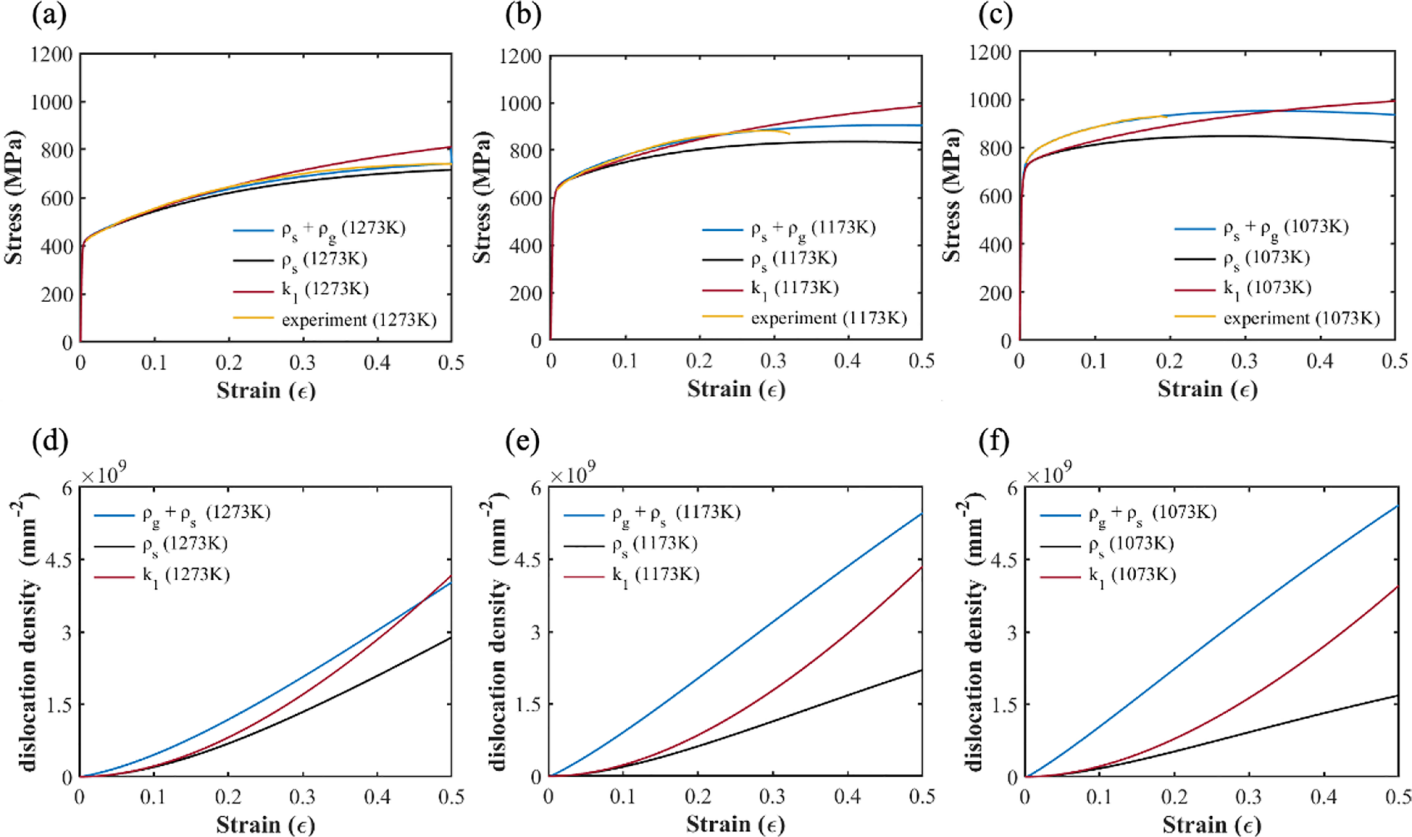
The stress–strain curves and total dislocation density evolution curves for the three hardening rules for the three annealing temperatures: (**a**) Stress–strain curve at 1273 K; (**b**) stress–strain curve at 1173 K; (**c**) stress–strain curve at 1073 K; (**d**) total dislocation density evolution curve at 1273 K; (**e**) total dislocation density evolution curve at 1173 K; and (**f**) total dislocation density evolution curve at 1073 K.

Figure 6a–c depict the simulated stress–strain curves of the different dislocation-based hardening rules and the experimental stress–strain curves for the three annealing temperatures, 1073, 1173, and 1273 K, respectively. It can be seen that the simulation results, which take into account both geometrically necessary dislocations and statistical storage dislocations, are consistent with the experimental measurements from Cho et al. Furthermore, Fig. 6d–f depict the evolution of the total dislocation density during the tensile tests for the samples. The blue, black, and red lines represent the three dislocation-based hardening rules, respectively.

In Fig. 6a–f, the difference between the black and blue lines represents the contribution of the geometrically necessary dislocation, and the difference between the red and black line represents the contribution of the dynamic recovery of the statistical storage dislocation. For the high annealing temperature of 1273 K, one can observe from Fig. 6a that the stress–strain curves of the three hardening models show little difference. Furthermore, one can observe in Fig. 6d that the statistical storage dislocation density is greater than the geometrically necessary dislocation density. It is thus determined that statistical storage dislocation primarily contributed to the strain hardening, whereas the geometrically necessary dislocation played a minor role. In other words, the contribution of precipitation strengthening post-yield can be neglected at high annealing temperatures.

On the other hand, for annealing temperatures 1173 K and 1073 K, it can be seen from Fig. 6e and f that the geometrically necessary dislocation density was larger than the statistical storage dislocation density. It can also be seen that the strain hardening behavior shown in the blue stress–strain curves is more obvious than in the black curves of Fig. 6b and c. Additionally, the stress–strain curves in Fig. 6b and c demonstrate softening behavior, whereas the total evolution curves of the dislocation density increase monotonically in Fig. 6e and f without saturation. Moreover, the results of our simulations indicate that the dynamic recovery of the statistical storage dislocations was significant for low annealing temperatures. Without the dynamic recovery term, the stress–strain curve was overestimated, as shown in Fig. 6a–c.

### Effect of particle size distribution on the strengthening mechanisms

**Figure 7:**
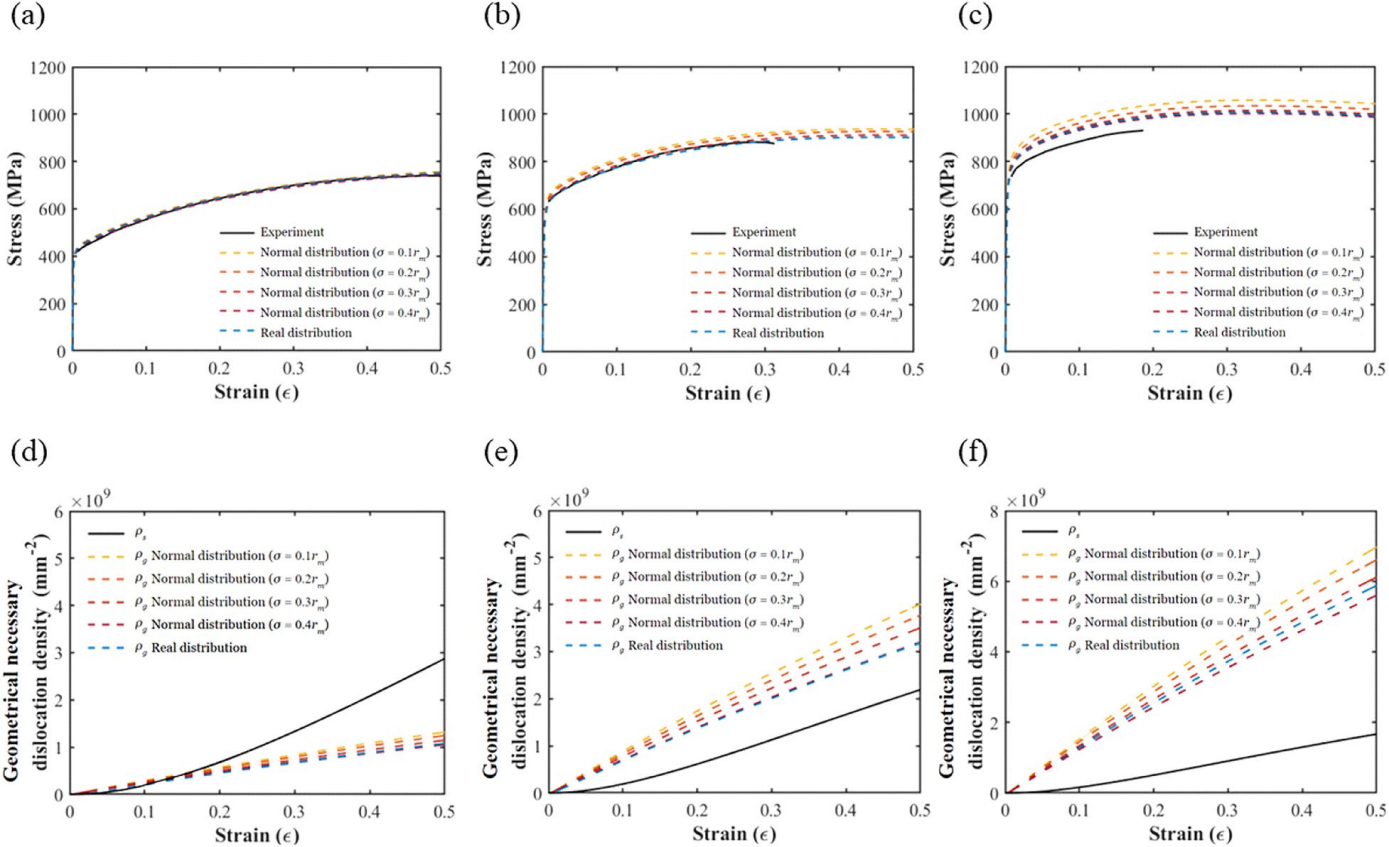
The stress–strain curves and dislocation density evolution curves for annealed samples with different precipitate size distributions: (**a**) stress–strain curves of 1273 K; (**b**) stress–strain curves of 1173 K; (**c**) stress–strain curves of 1073 K; (**d**) dislocation density curves of 1273 K; (**e**) dislocation density curves of 1173 K; and (**f**) dislocation density curves of 1073 K.

To investigate the effects of the precipitation size distribution on the strengthening mechanisms, we considered precipitation of different normal distributions that had the same mean value but different standard deviations. Figure 7 depicts the stress–strain curves and geometrically necessary dislocation density evolution curves of the different precipitation distributions at the three annealing temperatures from the numerical simulations. In Fig. 7a–c, the black and blue lines represent the stress–strain curves from the experimental data and from the numerical simulations based on the real precipitate distribution, respectively. The other lines represent the stress–strain curves from the numerical simulation results for precipitates with normal distributions.

From Fig. 7a–c, we can see that the stress–strain curves from the numerical simulation results based on real precipitate distributions are closer to the experimental results than are those based on normal distributions. This indicates that precipitate size distributions are important in determining the mechanical behavior of CoCrFeMnNi alloys. In addition, we can observe that the lower the annealing temperature, the larger the standard deviation of the precipitate size distribution, allowing the experimental data to be matched more accurately.

Figure 7a shows that the precipitate size distribution has little influence on the alloy stress–strain curve due to the smaller precipitate volume fraction at higher annealing temperatures, such as at 1273 K. On the other hand, it can be observed from Fig. 7b and c that in the case of 1073 K, the effect of particle size distribution on the mechanical properties of the alloy are more obvious with more precipitates. Moreover, we can see from Fig. 7d–f that the statistical storage dislocation density is independent of the precipitate size distribution. In contrast, the geometrically necessary dislocation density decreases with an increase in the standard deviation of the precipitate size distribution, which reduces the strain hardening in the stress–strain curve.

### Prediction of the stress–strain curve at different annealing temperatures

**Figure 8:**
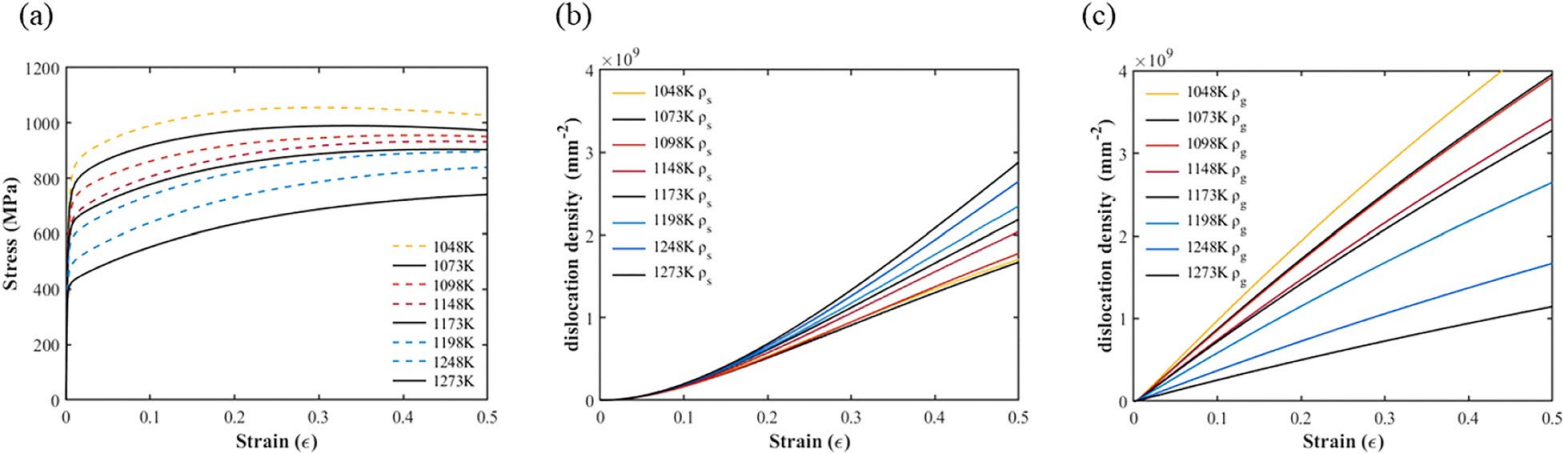
Predicted mechanical behavior of CoCrFeMnNi alloys at different annealing temperatures: (**a**) stress-stain curves; (**b**) statistical storage dislocation density evolutionary curves; and (**c**) geometrically necessary dislocation density evolutionary curves.

It would be desirable to know the mechanical behavior of CoCrFeMnNi alloys at different annealing temperatures. Thus, we can perform tensile test simulations of CoCrFeMnNi alloys for other annealing temperatures using the proposed computational framework according to the regression relationships between the model parameters and annealing temperature described in “Model parameters versus annealing temperature” section. The stress–strain curves and the dislocation density evolutionary curves for CoCrFeMnNi alloys with annealing temperatures from 1000 to 1300 K are shown in Figs. 8a–c, based on Eqs. (29)–(31). As expected, the lower the annealing temperature, the lower the statistical storage dislocation density and the higher the geometrically necessary dislocation density, which resulted in more obvious strain hardening stress–strain curves.

### Limitations of the proposed computational framework

We examined the mechanical response of the microstructures for different microscale parameters for various annealing temperatures through the proposed framework, and this enabled us to derive new insights into material design. We identified that that the particle distribution, controlled by the average size of the particle and the volume fraction of precipitation, can significantly enhance the strengthening effect. For a particle distribution close to a normal distribution with a smaller average size, we can expect high-strength and high-ductility HEAs. Although only the FCC-structured crystals and the σ-phase precipitate are discussed in this study, the proposed computational framework is not limited to dealing with a single-phase structure and a single type of precipitate. Dual-phase structures, such as FCC and BCC, can be directly simulated in our framework by setting the appropriate slip systems. On the other hand, different types of precipitates can be considered if these precipitates could be identified in SEM images.

This model can be easily extended to other alloy systems, which can be used by other researchers when designing new alloys with specific volume fractions of precipitation. To assess this framework, we have also provided the relationships for each temperature-dependent parameter. These relationships help us to understand the path from experimental measurement to mesoscale numerical studies. We envision that the distribution of precipitation dictates the growth of geometrical dislocation density by introducing a heterogeneous strain field.

Although the model enables us to study the mechanisms behind precipitates, there are some limitations that can be addressed in future work by expanding the experimental data to include more alloy systems with more annealing temperatures. Most of the experimental data used in the present study were from a single anisoatomic CoCrFeMnNi alloy. Incorporating more experimental measurements would help in the study of the hardening strategy of a complex alloy system, which often comprises a wide range of compositions. In addition, most of the annealing data was measured between 1073 and 1273 K. As such, incorporating higher annealing temperatures, which is an especially important limitation in the design of high-strength HEAs, would be challenging with the experimental measurements used.

There are several important strengthening mechanisms that could be studied using CPFEM-based modelling. Deformation twinning plays a crucial role in the mechanical properties of Cantor alloys. The increased ductility of Cantor alloys at low temperatures is thought to be due to the dynamic Hall–Petch effect, as twinning leads to more internal interfaces which act as Hall–Petch strengthening in polycrystalline materials^50^. The current framework can be extended to include twin density to account for deformation twinning.

Despite these limitations, the reported approach represents a powerful and efficient framework for the study of HEAs of a given annealing temperature. Our approach can be used to design new HEAs of tunable properties with a priori desired yield stresses and post-yield hardening. Furthermore, our presentation of dislocation density-based CPFEM will help inform the design of mechanically robust HEAs at desired annealing temperatures, especially given the vast design space of complex metal elements.

## Conclusions

A computational framework that integrated a theoretical model with the dislocation-based crystal plasticity finite element method was proposed to investigate the effects of precipitate distribution on the mechanical properties of CoCrFeMnNi alloys. SEM-BSE images of CoCrFeMnNi specimens with different annealing temperatures were analyzed using the ImageJ software to obtain the real precipitate distributions. Numerical simulations indicated that the precipitate distribution played an important role in the strain hardening behavior of the CoCrFeMnNi samples. In addition, the normal distribution, which had a standard deviation of 0.4 times the mean radius of the precipitate, provided an approximate precipitate distribution to efficiently predict the mechanical behavior of the CoCrFeMnNi specimens. The lower annealing temperature, which corresponded to a higher standard deviation of the precipitate size distribution, could fit the experimental data more accurately. The relationships between the model parameters and annealing temperatures were also regressed to predict the mechanical behavior of CoCrFeMnNi with other annealing temperatures. By studying the hardening effects from several mechanisms, we can distinguish precipitation hardening from other strengthening mechanisms as computed via the rate of dislocation density growth. This means that we can monitor the hardening that results from the distribution of different precipitated particles.

## Data Availability

The datasets used and/or analyzed during the current study available from the corresponding author on reasonable request.

## Code availability

The source code that supports the findings of this study are available on request from the corresponding author.
